# ZnO Nanostructures and Electrospun ZnO–Polymeric Hybrid Nanomaterials in Biomedical, Health, and Sustainability Applications

**DOI:** 10.3390/nano9101449

**Published:** 2019-10-12

**Authors:** Eloisa Ferrone, Rodolfo Araneo, Andrea Notargiacomo, Marialilia Pea, Antonio Rinaldi

**Affiliations:** 1 Department of Electrical Engineering, University of Rome Sapienza, 00184 Rome, Italy; eloisa.ferrone@gmail.com; 2 Institute for Photonics and Nanotechnologies–CNR, 00156 Rome, Italy; andrea.notargiacomo@ifn.cnr.it (A.N.);marialilia.pea@ifn.cnr.it (M.P.); 3 Sustainability Department, ENEA, C.R. Casaccia, Santa Maria di Galeria, Rome 00123, Italy

**Keywords:** ZnO nanostructures, toxicity, biocompatibility, physicochemical properties, cells viability assays, in vivo experiments

## Abstract

ZnO-based nanomaterials are a subject of increasing interest within current research, because of their multifunctional properties, such as piezoelectricity, semi-conductivity, ultraviolet absorption, optical transparency, and photoluminescence, as well as their low toxicity, biodegradability, low cost, and versatility in achieving diverse shapes. Among the numerous fields of application, the use of nanostructured ZnO is increasingly widespread also in the biomedical and healthcare sectors, thanks to its antiseptic and antibacterial properties, role as a promoter in tissue regeneration, selectivity for specific cell lines, and drug delivery function, as well as its electrochemical and optical properties, which make it a good candidate for biomedical applications. Because of its growing use, understanding the toxicity of ZnO nanomaterials and their interaction with biological systems is crucial for manufacturing relevant engineering materials. In the last few years, ZnO nanostructures were also used to functionalize polymer matrices to produce hybrid composite materials with new properties. Among the numerous manufacturing methods, electrospinning is becoming a mainstream technique for the production of scaffolds and mats made of polymeric and metal-oxide nanofibers. In this review, we focus on toxicological aspects and recent developments in the use of ZnO-based nanomaterials for biomedical, healthcare, and sustainability applications, either alone or loaded inside polymeric matrices to make electrospun composite nanomaterials. Bibliographic data were compared and analyzed with the aim of giving homogeneity to the results and highlighting reference trends useful for obtaining a fresh perspective about the toxicity of ZnO nanostructures and their underlying mechanisms for the materials and engineering community.

## 1. Introduction

**Figure 1:**
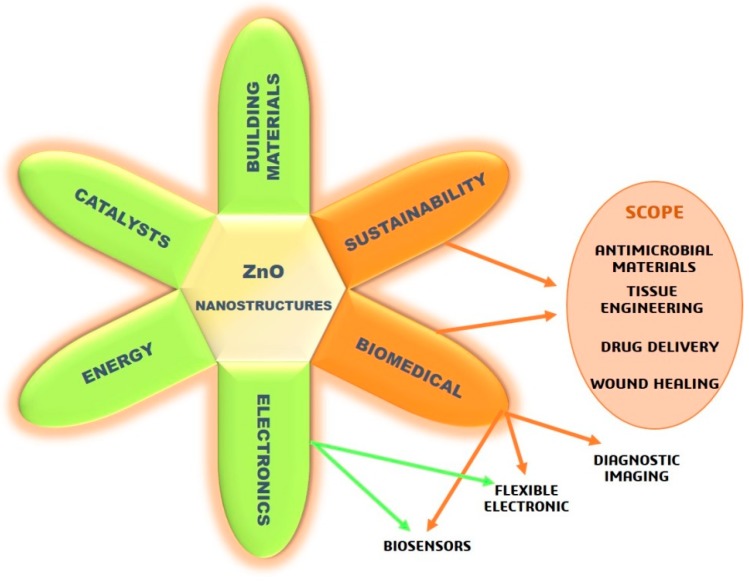
Applications of ZnO nanostructures, focusing on biomedical health and sustainability.

In recent decades, zinc oxide (ZnO) became an extremely popular in material science because of its multifunctional properties, low cost, and great versatility of use in various research areas and applications. The scientific interest was accompanied by a considerable growth of the ZnO market in industry, in sectors such as rubber, ceramic materials [1,2], paints [3,4], food packaging [5], cosmetics, and pharmaceutical products [6,7], as well as being highly used for electronic devices [8]. In addition, ZnO is recognized as a bio-safe material, and its use in cosmetic products is approved by the Food and Drug Administration (FDA), which is certainly a driving force in ZnO market growth. ZnO becomes all the more interesting in nanostructured form (i.e., shapes with at least one characteristic dimension less than 100 nm), enabling the realization of novel nanomaterials and nanodevices with special chemical–physical properties [9]. Moreover, it has the great advantage of easy synthesis with various techniques to achieve a vast group of nanostructures (NStr) including nanoparticles (NPs), nanowires (NWs), nanofibers (NFs), nanoflowers (NFls), nanorods (NRs), nanosheets (NSs), nanotubes (NTs), nanoribbons (NRBs), and tetrapods (TPs) [10,11,12,13,14], which suit best different given applications. The main application areas of nanostructured ZnO, summarized in Figure 1, range from electronics (with particular reference to flexible applications) to renewable energy and batteries, building materials, catalysts, and, not least, sustainability and biomedical applications. In fact, the use of nanostructured ZnO is highly increasing in the biomedical and healthcare sectors, allowing for diverse applications including antibacterial materials [15], tissue-engineering scaffolds [16], wound healing [17], drug delivery [18], molecular biosensors [19], and fluorescence imaging [20]. 

**Figure 2:**
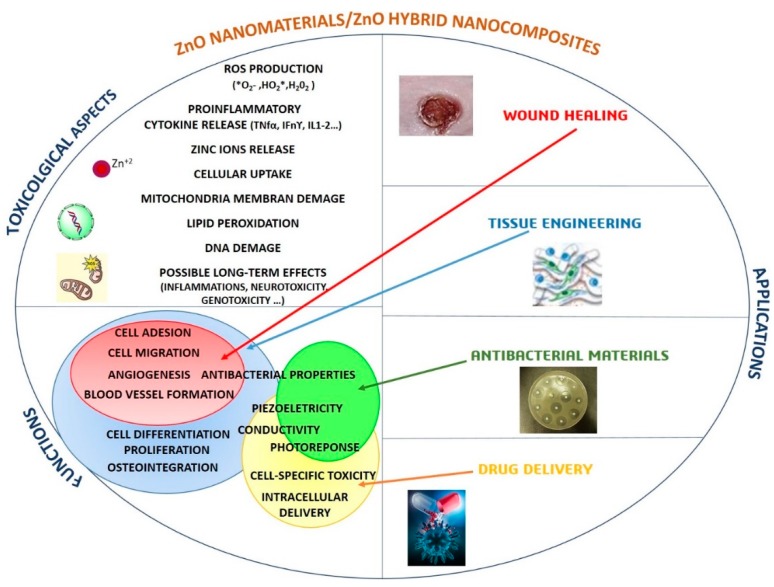
Toxicological aspects, functions, and scope of applications of ZnO-based nanomaterials and ZnO hybrid nanocomposites.

Since ZnO nanostructures are biologically very active because they can produce reactive oxygen species (ROS) and release Zn^2+^ ions [21], the development of new ZnO-based nanomaterials is indeed always accompanied by toxicological studies to test their biocompatibility. While still an open subject of research, numerous studies showed that the use of small amounts of ZnO NStr promotes cell growth, proliferation, and differentiation, as well as tissue regeneration, boosting angiogenesis and osteointegration processes, further supported by ZnO antibacterial and antifungal properties [22,23,24]. Moreover, ZnO NStr also present selectivity with respect to particular cell lines, which makes them potential candidates for killing cancer cells [25]. In addition to toxicological issues, the relationship between ZnO-based systems (materials and nanocomposites) and biological microenvironments is also particularly relevant for diverse target applications, as summarized in Figure 2, such that much effort is required at the materials design and manufacturing stage to ensure safe and effective application.

Within the manufacturing processes of novel biomaterials based on ZnO NStr, numerous methodologies use polymer matrices filled with metal oxides, to create high-performance hybrid composite materials [26,27]. Electrospinning is perhaps the most versatile and promising of these techniques, with low cost, ease of processing, and high scalability, which allows achieving two- and three-dimensional (2D and 3D) scaffolds of micro- and nanofibers [28]. This method makes it possible to produce large-scale continuous nanofibers which can be suitably set by acting on the process parameters [29]. The electrospinning technique allows the production of materials with controlled porosity and a large surface-to-volume ratio, which form an interconnected network suitable for biological applications, thanks to their morphological and mechanical properties [30]. The ZnO NStr can be used as fillers inside the electrospun polymer matrix or can be synthesized by post-processing polymer fibers, using hydrothermal processes or other techniques [31,32]. Finally, it is also possible to fabricate ZnO fibers by calcining the material after electrospinning process [33]. Biological processes are strongly influenced by material parameters such as fiber diameter, degree of porosity, shape and interconnection of pores, topography of the surfaces, homogeneity of dispersion of filling elements, and their concentration [34]. All these aspects can be appropriately set and monitored by acting on the electrospinning process parameters [35]. By combining the properties of the nanostructured ZnO with the ease of fabrication of the electrospinning technique, it is possible to develop innovative high-performance materials for many applications [36,37,38]. 

While many recent results about ZnO nanostructures and ZnO–polymeric nanocomposites manufactured by electrospinning exist for biomedical, health, and sustainability applications, the literature appears fragmented, and there is a need to critically investigate data trends and possible contradictions in the experimental data to boost further development in materials design and engineering. 

In this review, we focus on the use of nanostructured ZnO, both alone and within hybrid composite polymeric materials produced by electrospinning, for biomedical and sustainability applications. In Section 2 and Section 3, we reconsider the results of recent studies (in vitro and in vivo) on ZnO-based nanostructures with the aim of pointing out the underlying toxicity mechanisms, a preliminary aspect of fundamental importance for any safe deployment in engineering applications for life and environment. In Section 4, we report the synthesis approaches used for obtaining biosafe ZnO nanostructures for biomedical applications. Section 5 puts a spotlight on the influence of the chemical and physical properties of the ZnO nanostructures. Finally, in Section 6, we focus on ZnO–polymeric hybrid electrospun nanomaterials. Noteworthy, the effects of process parameters in electrospinning, such as different solvents, flow rates, needle-collector distance, etc., on fiber morphology and, to a certain extent, on biological performance are not in the scope of this review. 

## 2. Toxicity Studies on ZnO Nanostructures In Vitro

In this section, we report the main toxicity issues raised in the literature about ZnO nanostructures, as preliminary content for the subsequent survey of results and discussion later in this paper. We split the review and discussion into two subsections according to the presence of a large amount of data present on ZnO NPs which are considered first, before moving to other kinds of ZnO NStr.

### 2.1. ZnO Nanoparticles

The widespread use of ZnO NPs in many sectors was already highlighted; however, there are still open issues about their interaction mechanisms with biological systems, making this aspect still the object of study and research [39]. Hanley et al. [40] showed that ZnO nanoparticles induce a different cytotoxic response in primary human immune peripheral blood mononuclear cells (PBMC). In particular they found that lymphocytes are the most resistant cells, while monocytes are the most sensitive. All lymphocyte populations (cluster of differentiation 3-positive T cells (T-CD3), T-CD4, B) have a similar half maximal inhibitory concentration value (IC_50_; the concentration of drug/substance that is require for 50% inhibition in vitro) to all nanoparticle concentrations, while natural killer cells (NK) are more sensitive and show significant statistical differences at concentrations ranging from 1 to 5 mM; monocytes were even more sensitive with a mortality rate above 50% even at the lowest concentrations. It is important, in this regard, to consider the influence of physiological factors such as the electrostatic interaction between cell and nanoparticle, as well as intrinsic differences in the endocytosis/phagocytosis process. Other differences were found between native lymphocytes and memory lymphocytes, which could be linked to the fact that this second type of cells requires a lower level of activation of the threshold signal for proliferation, partly due to changes in the calcium level at the intracellular level. They also investigated the relationship between nanoparticle size and ROS production. What emerged is that cytotoxicity is inversely proportional to the size, with nanoparticles of 4 nm presenting the highest concentration of ROS value. Finally, they focused on the mechanisms of induction of immunoregulatory cytokines, a relevant factor to be considered for the potential use of ZnO NPs in biomedical applications. Their results showed that the induction of interferon gamma (IFN-γ), tumor necrosis factor alpha (TFN-α), and interleukin 12 (IL-12) is concentration-dependent, which is consistent with the production of oxidative stress and inflammation. This aspect indicates that the administration of ZnO NPs can elevate the production of important cytokines to stimulate a local response for an effective anti-tumor action. However, the potential damage from prolonged exposure to these cytokines should not be ignored and, hence, it is fundamental to control parameters such as size, concentration, and biodistribution of nanoparticles for their use in biomedical applications.

In other studies, Hanley and co-workers [41] showed that ZnO NPs induce cell-specific and proliferation-dependent toxicity, observing that rapidly dividing cells are more susceptible to ZnO toxicity with respect to quiescent ones. They highlighted the effects on tumor Jurkat cells and normal primary T-cells belonging to the same cell lineage and showed the differences between activated and resting T lymphocytes. The results showed that cancerous lymphocytes are about 25 to 35 times more susceptible to ZnO NPs than their normal counterparts. For this reason, the NPs can be designed to bind with antibodies, peptides, or small protein molecules associated with tumors, or they can be used for drug delivery. Moreover, the possibility of selectively eliminating activated T cells can be used for the treatment of autoimmune diseases such as multiple sclerosis and psoriasis, in which self-reactive T cells are one of the main groups underlying pathogenic processes. The inactivated state of these cells is verified by the lack of cluster of differentiation 40 L (CD40 L), a marker for T-cell activation. Finally, ROS production from PBMCs exposed to different concentrations of ZnO NPs and at different exposure times was investigated. Among these, monocyte cells ranked higher in the production of ROS. To evaluate the link between cell mortality and ROS production, T cells were pre-treated with *N*-acetylcysteine (NAC), a known ROS quencher. What emerged was that the use of NAC contributes significantly to preventing toxicity from ZnO NPs, indicating that the generation of ROS plays an important role in the toxicity induced by nanoparticles.

In the study by Heng et al. [42], the effects of spherical and sheet-like ZnO NPs on RAW-264.7 murine macrophages, BEAS-2B human bronchial epithelial cells, and mouse dendritic primitive cells (DC) were compared. Their choice to use human bronchial epithelial cells stemmed from the fact that the respiratory apparatus is often the first to come into contact with the polluted environment. Macrophages, on the other hand, are important components of the immune system, and it is, therefore, appropriate to study their interaction with the nanoparticles. Exposure of DC cells to ZnO NPs upregulates the expression of cluster of differentiation 80 (CD80) and cluster of differentiation 86 (CD86), known markers of DC activation and maturation, and stimulates the release of proinflammatory cytokines interleukin 6 (IL-6) and TNF-α, an aspect that emphasizes the potential role of ZnO NPs in inducing inflammation. For the experiments, spherical NPs and sheet-like NPs, with an average size of 20 × 20 nm and 325 × 15 nm, respectively, and concentration ranging from 1 to 30 µg/mL, were used. The results indicated a strongly dose-dependent behavior. The RAW-264.7 cell line appeared statistically more sensitive to spheriform particles than the BEAS-2B line, especially at high concentrations. This increased sensitivity may indicate that the cytotoxicity mechanism of ZnO NPs may involve the process of phagocytosis, or a second explanation could be that the ZnO NPs bind particular receptors of RAW-264.7 and activate apoptotic pathways. The differences in the cytotoxicity of the two forms could instead be due to differences in the dissolution rate. In any case, both forms stimulate the production of TNF-α with levels up to 200 times higher than the control, even at low concentrations (0.3 µg/mL). Finally, the associations of the ZnO NPs with both cell lines were evaluated, and the greater association occurred with the spherical nanoparticles, probably due to their smaller size. Moreover, the association was maximum after 4 h and then decreased. One root cause could be the process of exocytosis of the NPs after they are absorbed and accumulated inside the cells. The increased association of spherical NPs could make them more suitable for anti-tumor and drug delivery applications.

The same research group studied the cytotoxicity of ZnO NPs in BEAS-2B cells, highlighting the influence of oxidative stress in aggravating cytotoxic effects [43]. In that study, the cells were pre-exposed to 5 and 10 μM H_2_O_2_ for 45 min and subsequently exposed to variable concentrations of ZnO NPs with a size of about 10 nm. The results demonstrated an increase in cytotoxicity for cells pre-exposed to H_2_O_2_ with significant differences between those exposed to concentrations of 5 μM and those at 10 μM. The vitality test was not performed immediately, but 24 h later, as the activation of apoptotic pathways took some time to occur. It is to be noted that the cells exposed only to ZnO NPs had values of viability above 99% up to 10 µg/mL NP concentration, which then collapsed rapidly once exceeding this value. These data suggest the existence of a threshold value for the concentration of nanoparticles that does not compromise cell viability, a value that, however, decreases significantly in the presence of oxidative stress.

Wang et al. [44] studied the effects of toxicity and related action mechanisms on RAW 264.7 macrophage cells, in terms of cell viability, MTT (3-(4,5-dimethylthiazol-2-yl)-2,5-diphenyltetrazolium bromide) tetrazolium reduction assay, a colorimetric assay for assessing cell metabolic activity, mitochondrial membrane potential (MMP), total and released lactate dehydrogenase activity (LDH), intracellular ROS level, and Zn^2+^ ion concentration. The ZnO NPs were characterized in terms of morphology, size, surface charge, and solubility. The NPs had a polyhedral shape with an average diameter of about 37 nm. The average size in water was about 229 nm, according to the dynamic light scattering (DLS) measures, and, in some cases, there were aggregation phenomena due to a lack of surface protection. On the surface, they had a slight negative charge of −16 mV. In Dulbecco’s modified Eagle medium (DMEM), the dimensions increased significantly (about 1080 nm) due to the presence of salts that shielded the charge repulsion, allowing greater aggregation. Finally, in DMEM supplemented with 10% fetal bovine serum (FBS), there was a reduction in size (800 nm) due to the absorption of serum proteins, allowing a greater stabilization of the particles, in addition to a zeta potential increase to about −9 mV. Solubility studies at different pH values were performed to analyze the release of Zn^2+^ ions. What was found is that the release of ions in an acidic cell culture medium, namely, a phosphate-buffered saline (PBS) with 10% FBS (pH 5.5) that can be considered a lysosomal-mimicking medium, was of an order of magnitude greater than the value achieved in a culture medium at pH 7.2 (DMEM with 10% FBS). This result, in the authors’ opinion, is deemed reasonable because the acidic environment reached in these organelles releases H^+^ ions, which react easily with the ZnO to form Zn^2+^ and H_2_O. Cell viability studies showed time- and dose-dependent cytotoxicity, with decreasing viability as the NP concentration and incubation time increased. The study was performed for concentrations of ZnO NPs ranging from 25 to 200 µg/mL and incubation times of 4, 12, 24, and 48 h. The LDH activity was also measured. Those results were consistent with the MTT test, with LDH release increasing as incubation time increased, suggesting that cell membrane rupture is among the major causes of cytotoxicity. The MMP test was performed as an indicator of mitochondrial activity. MMP values of cells incubated with ZnO NPs decreased with incubation time, but at a faster rate than LDH values, which indicates that mitochondrial function is more compromised than cellular integrity. Finally, the concentration levels of intracellular Zn^2+^ and ROS were measured. There is still reason for uncertainty with regard to if Zn^2+^ ions are released into the culture medium and then transferred to the cell, or if the ZnO NPs are endocytosed and release the ions thereafter. Based on the results of their study, the authors concluded that the toxicity of ZnO NPs is linked to cell uptake and subsequent release of Zn^2+^ ions into the cytoplasm, particularly in organelles such as lysosomes with lower pH values.

Guo et al. [45] highlighted the molecular mechanisms involved in calcium homeostasis mediated by plasma membrane calcium ATPase (PMCA) by studying the cytotoxicity of ZnO NPs on rat retinal ganglion cells (RGC). After verifying, through MTT assay, the toxicity of high-concentration ZnO NPs toward RGC-5 cells, the authors chose three different concentrations (2.5, 5, and 10 µg/mL) for subsequent experiments. Through the study in the expression of PMCA2, cell membrane transport proteins responsible for the ejection of Ca^2+^ ions from the cytosol, they hypothesized the possible Ca^2+^-mediated signaling pathway, involved in the regulation of PMCA2 in the RGC-5 damage process caused by ZnO NPs. This mechanism hypothesized that ZnO NPs inhibit the activity of Ca^2+^ ATPase, increasing the levels of intracellular calcium ions and destroying intracellular calcium homeostasis, which in turn induces an overgeneration of ROS. The destruction of calcium homeostasis and the increase of ROS influence each other, leading to decreased expression of the PMCA2 gene and protein levels, thus initiating the apoptosis/necrosis mechanism of RGC-5 cells. The decrease in PMCA2 and protein levels was evidenced by the results of the quantitative polymerase chain reaction (Q-PCR) and enzyme-linked immunosorbent assay (ELISA) tests, which showed a decrease dependent on the ZnO concentration. The molecular mechanisms involved were also studied thanks to the help of real-time cell electronic sensing systems.

As noted above, the aerodigestive tract is considered to be particularly exposed to contact with NPs. In this regard, Moratin and co-workers [46] conducted a study comparing the cytotoxicity of malignant and non-malignant cell lines. They used human head and neck squamous cell carcinoma (SCCHN) derived from FaDu cells, chosen as a representative model of the mucosa of the upper aerodigestive tract, whereas they used human mesenchymal bone marrow stem cells (BMSCs) as non-malignant representatives. Cells were incubated at concentrations between 4 and 20 µg/mL for periods ranging from 1 to 48 h. The authors performed both the MTT assay and flow cytometry, in addition to fluorescence-activated cell sorting analysis (FACS), to improve the validity of the results. Both tests showed a reduction in cell viability, dependent on the ZnO NP concentration and exposure time. The mechanisms of apoptosis and necrosis appeared to be both responsible in the same way for cell death. Comparing the effects on the two cell lines, the applied doses of 5, 10, and 15 µg/mL were non-cytotoxic for the BMSCs, while the same concentrations diminished the cell viability of the FaDu cells, demonstrating the diversity of the effect on malignant and non-malignant cells. However, the comet assay revealed that, at low concentrations of 5 µg/mL, there was already significant DNA damage, even for non-malignant cells. In light of these results, the authors suggested a more critical and careful approach to the use of ZnO NPs in anticancer care.

After being absorbed at the respiratory, cutaneous, and gastro-intestinal levels, NPs can reach the blood and then migrate to different organs and systems, such as kidney, muscles, spleen, liver, and brain. When NPs come to the kidneys, they can impair metabolic functions and glomerular filtration; however, few studies are present on the toxicity of ZnO NPs on kidneys. A recent study by Reshna and Mohanan [47] focused on the in vitro toxicity of ZnO NPs on human embryonic kidney 293 (HEK 293) cells. The results showed a strongly concentration-dependent effect. From a morphological point of view, the transmission electron microscopy (TEM) analyses reported low changes at low concentrations, while, at 75 µg/mL ZnO NP concentration, the effects after 24 h of treatment were evident. To better consider the cytotoxic effects of ZnO NPs, the authors performed two independent studies: the MTT assay and the neutral red uptake assay (NR). The results showed a dose- and time-dependent toxicity. In the MTT assay, a net decrease in vitality was found at 25 µg/mL at each exposure time. The ROS concentration was measured, considered as the first mechanism of toxicity. Furthermore, changes were found in the actin distribution of HEK 293, depending on the dose of ZnO NPs used. Since actin filament is one of the critical elements in the cell division mechanism, such alterations in actin distribution can result in mitotic aberrations, leading to genomic instability. Changes were found in mitochondrial membrane potential, and lysosomal activity and the percentage of apoptotic cells were measured. Finally, through acridine orange staining (AO), autophagy (cell death type two) was determined, which is an important mechanism for the maintenance of cellular homeostasis, through the removal of damaged organelles, pathogenic organisms such as viruses and bacteria, etc. In addition, the appearance of some nuclear constrictions at high concentrations of ZnO NPs indicated dysfunctions in normal cell activity, due to DNA damage, leading to cell death by carcinogenesis.

**Figure 3:**
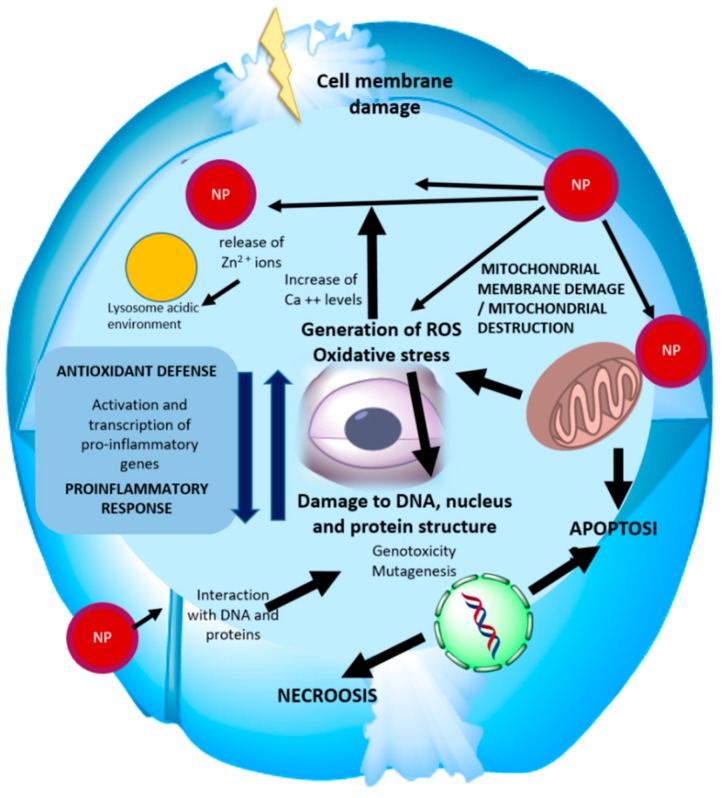
ZnO nanoparticle toxicity pathways mapped against a stylized cell.

In conclusion, from the results obtained in the aforementioned studies, it is possible to draw a synthetic scheme, reported in Figure 3, describing the mechanisms of interaction of ZnO NPs with the cell and the main causes of toxicity, explored further later.

### 2.2. Other Type of ZnO Nanostructures

In addition to the ZnO NPs, other hierarchical nanostructures acquired considerable interest in the biomedical field, thanks to their optic, optoelectronic, antibacterial, and detection sensitivity properties, as well as their selectivity with respect to particular cell lines, and the increase in cell proliferation and differentiation, which make them potential candidates for a new non-invasive approach for medical treatment [48,49,50,51]. The starting point, in order to open the way for future employment in the biomedical field of these NStr, is to understand how their morphological characteristics and physicochemical properties influence their toxicity and interaction with biological systems.

Paino et al. [52] investigated the cytotoxicity of flower-like nanostructures on Henrietta Lacks tumor cells (HeLa) and non-cancerous human fibroblast L929 cells, focusing on the effects of apoptosis and necrosis, ROS production, and cellular uptake. Cell viability tests were performed by incubating different forms of NFls for 24 h and at concentrations 0.1, 1, and 10 µg/mL, grown using a hydrothermal method at different times (4, 2, and 0.5 h). In particular, the NFls had rods of length between 1.7 and 2.3 µm and average diameters of about 250 nm. The results showed elevated cytotoxicity effects for HeLa cells, while the effects on non-carcinogenic cells were not statistically significant, showing that these nanostructures could be used for anticancer therapies without causing serious damage to healthy cells (in this case, fibroblasts). The morphological effects of NFls were also studied; the key parameter in determining differences in morphology was synthesis time. The internalization of NFls by HeLa induced cellular mortality by promoting oxidative-stress-dependent pathways, with increased intracellular ROS levels leading to necrosis.

Muller and co-workers [53] showed the effect of toxicity of ZnO NWs on human monocyte macrophages (HMMs) in cultures at similar concentration of ZnCl_2_, demonstrating that the release of Zn^2+^ ions is one of the main processes involved in toxicity. The NWs were synthesized by electrodeposition and had thicknesses of about 120–320 nm and lengths from 2 to 10 µm, depending on the duration of the electrodeposition process. Cytotoxicity was investigated by NR assay, measuring the accumulation of neutral red dye in the lysosomes of live cells. ZnO NWs with high aspect ratio in a ZnCl_2_ solution were incubated with the cells for 24 h at concentrations of 10, 20, and 40 µg/mL, showing similar toxicity, although only the concentration of 20 µg/mL showed statistically significant values. Confocal microscopy (CM) on cells confirmed an increase in the level of intracellular Zn^2+^ concentration before cell death. To better investigate the dissolution of the ZnO NWs, inductively coupled plasma mass spectroscopy analyses (ICP-MS) were performed in fluids at different pH values. They simulated a lysosomal acidic environment, as in the case of simulated body fluid-5 (SBF-5), and an environment mimicking the extracellular pH, and they recorded rapid dissolution in the first case compared to the second, demonstrating that dissolution firstly occurred inside the cells. Bright-field transmission electron microscopy (BF-TEM) showed a rapid uptake of the ZnO NWs. The HMM cells were incubated with 50 μg/mL ZnO NWs for 1 h; they showed a large phagocytosis of extensive aggregates of NWs. In conclusion, cell death presented features typical of apoptosis processes, such as condensation of chromatin and mitochondrial pyknosis, and of necrosis processes, such as plasma membrane rupture and leaching of cytoplasmic contents. The authors concluded that the ZnO NWs could be good candidates for drug-targeting, allowing modulation and control of the dissolution rate and delivery.

Gopikrishnan et al. [54] epitaxially grew ZnO NRs, with lengths of 50–60 nm and diameters of about 20–25 nm, using a hydrothermal method. They then evaluated their biocompatibility by analyzing their interaction with rat lung epithelial cells (LE). In particular, intracellular ROS levels were measured by studying the temporal kinetics of ROS production of LE treated with 2.5, 5, and 10 µg/mL ZnO NRs. After exposure, no increase in oxidative stress or lipid peroxidation was observed in the cells exposed, even at the highest periods of time and concentration levels. The MTT cell viability test showed independence of the viability of the LE cells relative to concentration and exposure time, always above 95%. These results, according to the authors, were due to the low concentration of released Zn^2+^ ions. Therefore, the hypothesis of existence of a threshold limit value for the concentration of Zn^2+^ that induces toxicity, a value never reached in their experiments, was established.

A study by Ahmed et al. [55] highlighted the mechanisms of apoptosis and oxidative stress in human alveolar adenocarcinoma cells (A549) caused by ZnO NRs. The high level of antioxidant enzymes superoxide dismutase (SOD) and catalase (CAT) suggested that oxidative stress was one of the main mechanisms of toxicity. The results of the atomic absorption spectrometry confirmed the release of Zn^2+^ ions, showing that a concentration of 10 μg/mL Zn^2+^ was released for cultures with 100 µg/mL ZnO NR concentration. However, this concentration of Zn^2+^ proved to be not particularly cytotoxic for the A549 cells, a result that was consistent with previous studies that showed that these concentrations of Zn^2+^ are not sufficiently high to cause cytotoxic effects in human cells, unless there is a contact between the particles and the cells. The emergence of apoptotic processes was demonstrated by the fact that ZnO NRs upregulated the cell-cycle checkpoint protein p53, a transcription factor that regulates the cell cycle and can initiate apoptotic processes, and pro-apoptotic Bax protein, while downregulating proteins antiapoptotic survivin and Bcl-2. Furthermore, ZnO NRs induced the activity of caspase 3 and caspase 9, enzymes that play a fundamental role in the phases of cell apoptosis. All these results suggested that ZnO NRs induced apoptosis and oxidative stress in the A549 cells via p53, survivin, Bax, Bcl-2, and caspase 3 pathways.

Wang et al. [56] analyzed the biological effect of an array of densely packed and vertically aligned ZnO NWs on three types of excitable cells, the NG108-15 cancerous neuronal cell line, the HL-1 cardiac muscle cell line, and neonatal rat cardiomyocytes. MTT assays showed a statistically significant inhibitory effect by ZnO NWs on mitochondrial activity after one day of culture, especially on NG108-15 and HL-1 cells, compared to gold, glass, and polystyrene substrates. The NG108-15 cells, HL-1 cells, and cardiomyocytes had diameters of about 10–100, 20, and 13 μm, respectively, while the thickness of the NWs was about 300 nm. Every single cell was completely covered by the NWs. The inhibitory effect could be due to the penetration of the cell membrane by the NWs or the lack of adhesion of the cells to the substrate. In the first case, the penetration of the NWs into the membrane would be strongly influenced by their diameter; instead, in the second case, the engraftment failure could be due to the topography of the array, in particular the density and spacing of NWs, which can lead to an insufficiency in flatness of the surface required for cell adhesion. Another factor of toxicity could be the release of intracellular Zn^2+^ ions in the acid environment of lysosomes. This hypothesis is not, however, among the most probable because the NWs are fixed to a substrate and, therefore, the process of phagocytosis could be difficult. Another interesting aspect that emerged from this study is that primary cardiomyocytes seem to better tolerate the inhibitory effect of NWs. This result was consistent with previous studies on NPs that showed a selectivity to cytotoxicity on rapidly dividing cells compared to primary cells. The authors concluded that the biocompatibility of ZnO NWs can be raised, but it is difficult to do it in densely packed arrays. 

Papavlassopoulos et al. [57] tested the biocompatibility of ZnO TPs on human dermal fibroblasts (NHDF) by highlighting the influence of cell culture conditions and material properties on cytotoxicity. They found that the toxicity of TPs was significantly lower than that of spherical NPs. Furthermore, the morphology of ZnO TPs influenced cellular toxicity in contrast to surface charges modified by UV light illumination or O_2_ treatment and material age. Finally they observed that the direct contact of the material with the cells had greater toxicity than the transwell culture models that caused only an indirect effect through the released of zinc ions.

## 3. Toxicity Studies on ZnO Nanostructures In Vivo

**Figure 4:**
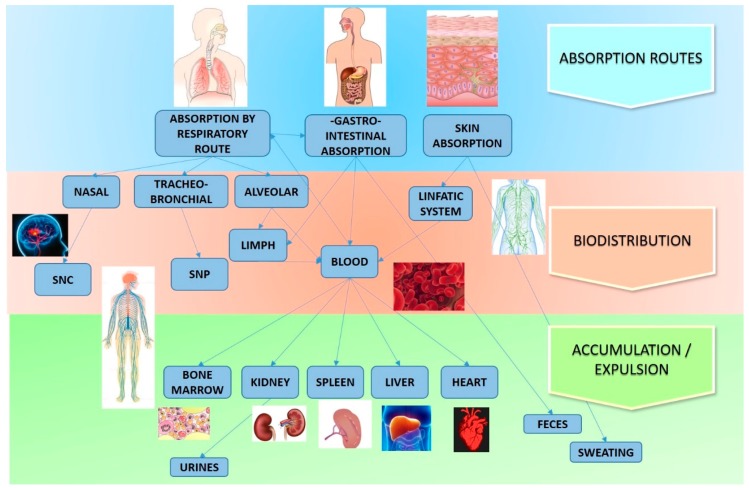
ZnO nanostructure (NStr) interaction mechanisms with the organism (SNC: central nervous system; SNP: peripheral nervous system).

In the previous paragraphs, the effect of the ZnO NStr on different cell lines in vitro was described. Here, we focus on the studies that were carried out in vivo to evaluate the interaction with organs and apparatus. Figure 4 describes the main routes taken by ZnO NStr, starting from absorption, up to their distribution and accumulation or expulsion.

Before starting with the description of recent in vivo studies, it is necessary to make a series of considerations in order to have a more conscious and focused approach for the analysis of the results obtained, as listed below.

- Most of the experiments were carried out on laboratory animals;
- In most cases, the doses were administered at one time and the concentration was significantly higher than the actual exposure conditions;
- There was no analysis of the long-term effects on the organism;
- There was no long-term study evaluating the effects due to exposure to small systemic concentrations.

Therefore, further analyses are required to have a comprehensive view of the effects of the ZnO NStr, looking for long-term effects and small exposure doses. However the results of the studies examined in this section provide a useful starting point for the assessment of risks associated with exposure to these nanomaterials. 

Tian et al. [58] investigated the effects of neurotoxicity induced by ZnO NPs on differently aged mice, by studying the interaction between age and exposure to nanoparticles. According to the life cycle of CS7BL\6J mice, mice aged six months have a psychological age comparable to that of a 30-year-old man, while mice of 18 months can be compared to a man of 56 years; in both cases, we refer to healthy specimens. Firstly, the authors showed that ZnO NPs induced a systemic inflammatory response in both categories of mice, but with more severe effects in older mice, with a synergistic effect between the age of mice and exposure to NPs, with increased production of proinflammatory cytokines IL-1 and IL-6 in the blood. These data indicated that older individuals exhibited more severe inflammatory disorders during exposure with ZnO NPs. The neurotoxic effects were studied by intraperitoneal injection of 5.6 mg/kg ZnO NPs. Also, in this case, there was an increase in proinflammatory cytokines in the brain, with the same synergistic effect between age and exposure to NPs. In addition, significant increases in SOD and glutathione peroxidase (GSH-Px) concentration levels and increased malondialdehyde (MDA) concentration were observed, indicating oxidative stress conditions, especially in older individuals. The authors also analyzed the neurocognitive functions of mice. Data showed that long-term memory and passive avoidance ability were impaired following exposure to NPs, particularly in older mice. However, no significant changes in motor activity and in the exploratory behavior of mice were recorded. Instead, damage to spatial cognition was found, suggesting a link with the potential of the hippocampus, strongly linked to cognitive learning and memory skills. These dysfunctions are probably related to systemic inflammation of the central nervous system (CNS), which was further investigated by analyzing the levels of hippocampal proteins CREB and P-CREB, which decrease in age in a quantity-dependent manner. The authors stated the importance of the results obtained, although the doses were administered at one time and with high concentrations, with NPs of a single size. It is, therefore, essential to carry out further studies analyzing the effects of NP size and to evaluate chronic exposure at low concentrations.

Ansar et al. [59] examined the effects of hesperidin (HSP), 100 mg/kg body weight (bwt), on ZnO NPs during oral administration of 600 mg/kg bwt ZnO NPs in rats. The effects of neurotoxicity induced by NPs were evidenced by the increase in inflammatory markers, including TNF-α and proinflammatory interleukins. Furthermore, increases in C-reactive protein (CRP), CAT, GSH-Px, and glutathione (GSH) were recorded in the brains of rats, linked to the oxidative stress response. The administration of bioflavonoids such as hesperidin may play a protective role, inhibiting the induction of antioxidant enzymes and improving the ZnO NP-induced neurotoxicity. In fact, the results indicated a significant decrease in the levels of inflammatory cytokines in the blood of rats.

Liu et al. [60] studied the in vivo effects of the ZnO NPs on neuronal factors and on the neuroendocrine cells of the ovaries. For the study, concentrations of 25, 50, and 100 mg/kg (diet) ZnO NPs were used to treat pubertal hens. It was found that, at concentrations of 50 and 100 mg/kg, the ovarian organic index slightly decreased. Furthermore, the data indicated that the concentrations of essential elements in the ovaries, such as Zn, Fe, K, Ca, and Mn, increased, especially following exposure to the highest ZnO NP concentrations. The data showed that the increase in the concentrations of these elements was related to the levels in the genetic and protein expression of Neural Cell Adhesion Molecule 1 (NCAM1), Doublecortin (DCX), Roundabout Guidance Receptor 1 (ROBO1), Choline O-Acetyltransferase (CHAT), and neurofilament heavy (NF-H). The authors used quantitative transcriptomics (RNA-seq) to determine the effects of ZnO NPs on the gene expression of ovarian samples. It was found that 222 genes were modified by the treatment with ZnO NPs at 100 mg/kg, and, of these genes, 32 were related to neuronal factors (including those mentioned above) that are very important for organ development. To clarify the still unclear biological effect exerted by the ZnO NPs, or by the Zn^2+^ ion release, the authors used ZnSO_4_ to compare these effects. They concluded that, in their study, both the ZnO NPs and the Zn^2+^ ions exerted their action on the biological system, since the ZnO NPs produced both effects similar to ZnSO_4_ and specific effects, such as those on the regulation of neuronal factors in protein and gene expression.

Regarding the cytotoxic effects exerted by the ZnO NStr on the reproductive system, Han et al. [61] investigated the effects of ZnO NPs in vitro and on male mice. They studied cytotoxic effects in vitro on Leydig cells (LCs) and Sterol cells (SCs), and in vivo, via injection of a single dose on CD1 mice. LCs and SCs are two cell lines essential for the development of the gonads and for spermatogenesis; LCs play a fundamental role in the synthesis of steroidal testosterone, in sperm maturation, and in sexual functions, while SCs are located in the seminiferous tubules of the testes and provide nourishment, as well as structural and morphological support for germs during spermatogenesis. The results showed toxicity following the internalization of cell lines with ZnO NPs, manifested with apoptotic phenomena related to DNA damage and loss of mitochondrial membrane potential induced by ROS increase. In the in vivo tests, the authors observed significant reductions in the thickness of the seminiferous epithelium and in the diameter of the seminiferous tubules, in mice treated with a single injection of 5 mg/kg ZnO NPs. Furthermore, a statistically significant percentage of sperm showed morphological alterations such as double head, small head, double tail, etc. 49 days after treatment with ZnO NPs, with possible consequences on the fertility of mice.

Another organ in which the nanoparticles tend to accumulate is the kidney, causing toxicity on the cells and compromising important vital factors. Xiao et al. [62] focused on the toxic effects of ZnO NPs in vitro on podocytes and in vivo on rats. Variations of 10, 50, and 100 µg/mL were used to perform in vitro studies on podocytes, which showed induction of apoptosis by increasing intracellular ROS generation, as also confirmed by an experiment in which cells treated with *N*-mercaptopropionyl-glycine, known an ROS scavenger, showed decreased levels of apoptosis following exposure with NPs. The MTT assay revealed that the viability of the podocytes decreased in a dose- and time-dependent manner; in particular, a concentration of 100 µg/mL ZnO NPs caused a dramatic reduction in cellular activity. In in vivo acute toxicity studies, adult male Wistar rats were treated with 3 mg/(kg∙day) ZnO NPs for five days. Decreases in important vital factors such as body weight and kidney index of rats were recorded, which suggested a potential toxicity of ZnO NPs on the kidney. Furthermore, the data showed a significant reduction in CAT and SOD levels, indicating an evident disturbance of the antioxidant functions, a result consistent with in vitro experiments. Finally, the loss of important proteins such as nephrine, a fundamental protein for the correct functioning of the renal filtration barrier and a structural component of the podocyte filtration barrier, was diagnosed. The latter aspect suggests to the authors that the ZnO NPs can interfere in the process of protein synthesis.

Concerning the genotoxic effects on different organs, Bollu et al. [63] assessed the effects of in vivo genotoxicity on Swiss Albino mice subjected to a dose ranging from 0.5 to 6 mg/kg of rod-shaped ZnO NPs of approximately 18 nm, administered orally for seven consecutive days. Their results showed no genotoxicity and no toxicity to the liver, heart, kidney, or spleen. In particular, the micronucleus assay was performed, a test to verify the formation of micronuclei during cell division processes and to indicate the presence of genotoxicity and chromosomal instability. The results of these tests showed the same percentages in the number of polychromatic erythrocytes in all the involved groups and compared to the control, indicating a non-dose-dependent effect. The chromosome aberration assay also demonstrated a non-significant increase in chromosomal aberration and the absence of chromosomal damage. Also, in this study, no percentage changes in the mitotic index were found, suggesting no incidence on cell proliferation. The alkaline comet assay reported no DNA damage caused by the ZnO NRs; in fact, there were no significant changes in the length of the comets. The results obtained are inconsistent with those of other research groups. The authors attribute these differences to the following possible motivations:

(1) The incidence of the shape and size of the particles; in fact, other studies reported that spherical and smaller nanoparticles are more likely to be taken up;
(2) The use, in this study, of small doses, comparable to those used in clinical procedures, which were much lower than those generally used in literature;
(3) The difference in conditions between in vitro and in vivo studies. The authors stressed the importance of the results obtained, but also the importance of carrying out further studies considering different routes of exposure, such as dermis, inhalation, etc.

## 4. New Approaches to Synthetize Safe ZnO Nanostructures for Biomedical Applications and Cancer Therapy

Numerous efforts are being made to synthesize biocompatible and safe ZnO nanostructures, suitable for use in the biomedical field. In this regard, Lewiński and his team [64] used a new organometallic self-supporting approach to synthesize “safety by design” ligand-coated ZnO nanocrystals (NCs). ZnO NCs of high quality and with size down to a quantum regime (<7 nm) were coated with densely packed 2-(2-methoxyethoxy) acetic ligands (MEAA), to obtain ZnO–MEAA NCs with average core size and hydrodynamic diameter of 4–5 nm and 12 nm, respectively. The characteristics of the nanocrystal–ligand interface, which gave protection to the core through an impermeable shell and a well-passivated surface, strongly influenced the physiochemical properties and biocompatibility of these nanostructures. In fact, in vitro cytotoxicity studies on normal human fetal lung fibroblast cells (MRC-5) and human lung cancer cells (A549), performed with MTT assay, showed low toxicity compared to structures of the same size synthesized with traditional methods, such as wet chemistry. In particular, even at the highest concentration tested of 25 µg/mL, the effects of toxicity were lower than the data in the literature about particles of the same size, despite using cell lines considered among the most sensitive. In addition, the ROS generation tests and the Zn^2+^ ion concentration reported relatively low values, indicating that the organometallic procedure conferred a good waterproof protection through the organic ligand shell, which inhibited the loss of Zn^2+^ ions by the core, improving surface stability. The authors conclude that this method can open new frontiers for the design of new, safe ZnO-based materials for biomedical applications.

Chun et al. [65] prepared zinc aminoclays (ZnACs) with functionalized primary amines ((–CH_2_) 3NH_2_) via a sol–gel reaction, and studied their in vitro toxicity on HeLa cells and in vivo toxicity in zebrafish embryos. The purpose of their study was to compare the ZnACs with ZnCl_2_ and Zn(NO_3_)_2_ salts and with ZnO NPs. In vitro studies on HeLa showed greater toxicity of ZnACs, probably caused by their greater bioavailability and uptake, as well as their positively charged hydrophilia caused by the production of ROS, especially in the case of ZnACs in their form of cationic nanoparticles. For in vivo toxicity analyses, the authors studied the duration of embryonic development at hatching in zebrafish exposed to ZnACs and ZnO NPs for 72 h. In both cases, a dose-dependent inhibition of embryo hatching was found. However, the ZnO NPs proved to be more toxic, probably due to their aggregation characteristics, colloidal behavior, and smaller hydrodynamic dimensions. However, the ZnACs reported toxicity effects on zebrafish embryos at the highest concentrations of 50 and 100 µg/mL.

ZnO nanostructures are proving very promising in drug delivery for the treatment of tumors, exploiting the selective effect of toxicity of ZnO particles toward the diseased cells, minimizing the impact on healthy cells. Zeng et al. [66] studied a lymphatic targeting drug delivery system for the treatment of lymphatic metastatic tumors, using lipid-coated ZnO NPs (LZnO-NPs). They synthesized core–shell nanoparticles (30 nm) loaded with 6-mercaptopurine (6-MP) using a water-in-oil (W/O) microemulsion. The MTT assay demonstrated the high selectivity of LZnO-NPs to cancer cells. In addition to the acid-sensitive behavior, an effective internalization of the particles in the cancer cells was enabled, with a rapid release of the drug in the cytoplasm and ZnO decomposition in the acid environment of the lysosomes. These results were also demonstrated by the acid sensitivity release experiment which indicated a prolonged drug retention time in the blood circulation (pH 7.4) and a rapid release in the lysosomes when the particles are internalized by the cells. The measurement of ROS levels showed a non-significant increase in primary lymphocytes due to their antioxidant capacity, which does not occur in cancerous cells where there is significant ROS accumulation. In vivo tests on Sprague-Dawley rats confirmed the higher biocompatibility of LZnO-NPs, compared to non-coated ZnO NPs; the red blood cell (RBC) tests showed that the RBCs did not aggregate in the presence of LZnO-NPs and there was no blood hemolysis, unlike the ZnO NP test. Biochemical parameters in the liver showed reversible hepatotoxicity in the case of LZnO-NPs, with parameters returning to control values, whereas hepatotoxicity was non-reversible in the case of ZnO-NPs. Histopathological analyses showed no significant lesions in the organs analyzed in the case of LZnO-NPs, while, in the case of ZnO-NPs, they showed mild–moderate inflammation in the intestine, kidneys, and lungs; LZnO-NPs only caused slight congestion in the spleen during the first hours of administration.

A new approach to breast cancer treatment was proposed by Vimala and his group [67]. They exploited the synergistic effect between chemo-photothermal targeted therapy and a multifunctional drug delivery system, developed through biosynthesis of polyethylene glycol (PEG)-coated ZnO nanosheets modified with folic acid (FA) after aminic functionalization (FA–PEG–ZnO NS), loaded with doxorubicin (DOX). The best results were found in the case of combined therapy, compared to that which exploited only the photothermal effect or only chemotherapy. The cancer cells in fact showed a good uptake and an effective internalization of DOX–FA–PEG–ZnO NS; in addition, under near-infrared irradiation (NIR), the maximum toxicity toward the breast cancer cells was found with respect to other cells and to the control. In vivo toxicity to mice was also tested, with results confirming the biocompatibility of the particles; a slight toxicity was observed for the liver linked to ROS production, while histopathological and morphological analyses on kidneys, lungs, brain, heart, and testes did not show abnormalities for these organs. Biocompatibility was also tested by injecting DOX–FA–PEG–ZnO NS through the tail vein, and the post-injured mice showed no pathology, and hematology markers did not show significant alterations.

## 5. Influence of the Chemical and Physical Properties of the ZnO Nanostructures on Toxicity 

A key question regards the main mechanisms of toxicity induced by ZnO NStr, trying to understand the incidence of chemo-physical and morphological properties, such as size, shape, surface area, surface charge, and surface functionalization, as well as distribution, concentration, and aggregation phenomena. Reported studies showed that the main causes of cytotoxicity include the production of ROS (with consequent oxidative stress and lipid peroxidation), zinc ion release, the breakdown of the cell membrane, the impairment of mitochondrial functions, and DNA damage [40,41,42,46]. The results on the release of LDH confirmed that cellular rupture is among the main causes of toxicity, even if the results of MMT tests suggest that the mitochondrial functions are even more compromised [44]. The ZnO NStr also induce the production of immunoregulatory cytokines, which, on the one hand, can stimulate a defense response, and, on the other, they can cause long-term undesirable effects [41,58]. Other cytotoxicity mechanisms involve calcium homeostasis processes, as the ZnO NPs inhibit the activity of Ca^2+^ ATPase [45]. Furthermore, alterations in the expression values of some genes and protein levels were found to trigger apoptosis/necrosis mechanisms leading to cell death. Even alterations in cell division processes can lead to genomic instability [45,55].

With regard to the sensitivity of the different cell lines, considerable differences were observed. Among PBMC cells, the lymphocytes proved to be the most resistant cells, while the monocytes were among the most sensitive [40]. Moreover, within the lymphocytes, there were differences between native lymphocytes and memory lymphocytes, with proliferation-dependent toxicity levels. The rapidly dividing cells were more susceptible to the toxicity of the ZnO particles; this selectivity of the ZnO nanostructures makes them interesting in future perspectives for cancer treatment [41]. To confirm this, it was shown that the cytotoxic effects of ZnO are different depending on whether malignant and non-malignant cell lines are studied; the former are in fact more affected by the toxic effects of the particles [46,52]. Considering the different contact routes and the biodistribution of nanoparticles, the upper aerodigestive tract is considered one of the regions most affected by exposure to nanoparticles; in particular, the respiratory apparatus cells are among the first to come into contact with the ZnO NStr, which can in fact be easily inhaled [42,43]. Kidney cells also appear to be compromised in terms of metabolic and filtration functions.

The concentration of nanostructures is another crucial parameter for the determination of their toxicity, and it was shown to be concentration- and time-dependent [40]. Cell viability, in fact, decreases with increasing concentration and incubation time [43,44,46]. However, some other studies supported that the mechanism of association is maximum in the first hours of interaction, before stabilizing or, in some cases, reducing, possibly due to exocytosis phenomena [42]. Many studies highlighted the possible existence of a maximum threshold value for the concentration of ZnO NStr that does not compromise cell viability and, beyond which, the effects of toxicity increase as a function of the increase in concentration [43,54]. For the ZnO NPs, this value is around 5–10 µg/mL [43].

**Figure 5:**
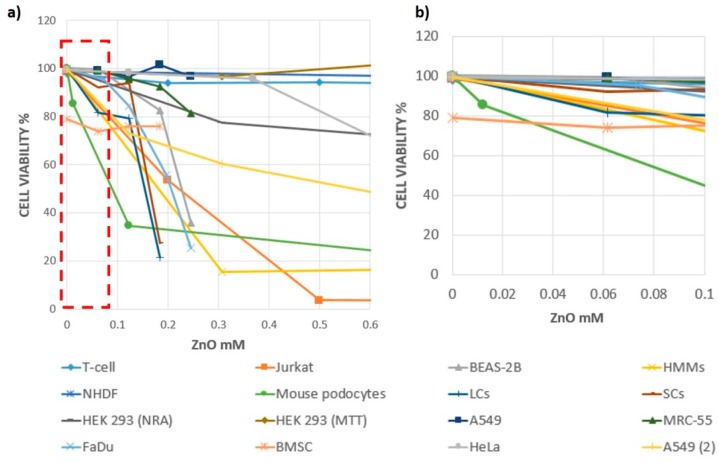
(**a**) Overall elaboration from published data about cell viability vs. ZnO concentration, for different cell lines and different types of nanostructures; (**b**) zoom-in of the red dashed area in (**a**), highlighting the ZnO concentration range where cell viability is preserved and is relatively “safe”.

Figure 5 shows the vitality values for different cell lines as a function of ZnO concentration and considering 24 h of exposure; the values were extrapolated from the results of some of the studies described in the previous paragraphs. The trends obtained are purely qualitative and refer to extremely heterogeneous data that were affected by the variability of the experimental methodology, the different culture conditions, the density of cells used, and numerous other factors that make the comparison between the data not easy to interpret. However, useful general considerations can be made allowing a global overview of the results. In particular, in Figure 5a, the cellular vitality values normalized with respect to the initial cell density are reported in the ordinate, and the ZnO concentration is reported in the abscissa, expressed as molarity. The analyzed data are summarized in Table 1, where the value of the initial cell density, the type of culture plate, the type of vitality test, and the type and dimensions of nanostructure are indicated for each experiment. Observing the trends, we can appreciate the previous observations relating to the resistance of the different cell lines, as well as the influence of the concentration and the type of nanostructure examined. Figure 5b shows an enlargement of the ZnO concentration range, between 0 and 0.1 mM, i.e., from 0 to about 8 µg/mL, within which all the cell lines considered, with the exception of podocytes (which have limit values of lower concentration), show viability values above 70%, regardless of the type of nanostructure.

Relative to the influence of the size, a correlation was found between the production of ROS and the nanostructure size, with cytotoxicity inversely proportional to the size of the particles, whereby the smallest particles presented the highest levels of ROS concentration [40,42]. In addition, small particles are more likely to be involved in engrafting phenomena and subsequent phagocytosis. On the other hand, the nanostructures shape affects the dissolution rate. Solubility at different pH values modulates the release of Zn^2+^ ions; in particular, the release of ions in acidic culture environments, mimicking the lysosomal one, presents much higher values than those obtained in environments with a neutral pH [44,53]. Nanostructures of different shapes have different cytotoxic effects. Small and spherical nanoparticles exhibit the highest levels of ROS concentration; moreover, the greater association of the ZnO spherical nanoparticles makes them more suitable for anticancer and drug delivery treatments [42]. The ZnO NPs can in fact be designed to combine with antibodies or small molecules, peptides, or proteins associated with tumors and be destined for drug delivery applications. The ZnO NFls present different toxicity to cancer cells than healthy cells, with levels in the apoptotic indicators much higher in diseased cells, characteristics that make them suitable for anticancer applications [52]. The ZnO NWs present high dissolution values and have a tendency to aggregate; moreover, like the ZnO NPs, they manifest a rapid uptake and can be phagocytized, initiating apoptotic and necrotic processes [53,54]. They are also among the potential candidates for drug-targeting applications [53]. ZnO NRs have lower levels of toxicity with higher threshold values than other nanostructures; however, at sufficiently high concentrations, they can initiate apoptotic processes and lead to oxidative stress [54,55].

Despite the great importance of the results obtained in vitro, the in vivo approach is very different and more complex, due to the increased number of variables involved. The absence of an official protocol, the diversity in the selected concentrations, which are often administered in a single dose and with much higher values than the clinical ones, the great complexity of the biological interactions, and the variability of the physiological boundary conditions make the interpretation of the results obtained in tests on animal models an extremely complex process [58,61,62,63]. According to the in vitro results, increases in SOD, MDA, and proinflammatory cytokine production were also measured in in vivo tests [58]. Neurotoxicity studies showed evidence of long-term memory impairment and spatial cognition, probably caused by systemic inflammation of the central nervous system, as evidenced by the measured levels of hippocampal proteins and the increase in inflammatory markers [58]. Studies on neuroendocrine cells highlighted alterations in the concentration of essential elements in the ovaries, linked to levels in gene and protein expression [60]. The reproductive system of male mice presented morphological changes in the sperm, including reduction of the thickness of the seminiferous epithelium and the diameter of the seminiferous tubules [61]. Harmful effects caused by exposure to ZnO NPs were also found in the kidneys, with reduction of important vital factors such as kidney index and degradation of the filtering functions [61]. Other organs subjected to the toxicity of the nanoparticles are the liver, spleen, pancreas, lungs, and brain. However, studies on ZnO rod-shaped nanoparticles reported non-significant effects of toxicity in the liver, kidney, spleen, and heart, in addition to the absence of genotoxicity, confirming some results of in vitro studies that showed the lower toxicity of this type of nanostructure [63].

Some new research lines focused on the development of Zn–ZnO nanostructures, engineered with particular functional groups, core–shell coated, hybrid organic–inorganic, often obtained through the use of biosynthesis methods, with the aim of increasing their biocompatibility or enhancing some properties and strengthening their use in the biomedical field and in oncology. The results in vitro and in vivo showed the greatest biocompatibility of these constructs, which have lower levels of toxicity compared to the traditional ZnO NStr [64,65,66,67].

## 6. ZnO–Polymeric Hybrid Electrospun Nanomaterials

### 6.1. Tissue-Engineering Applications

Tissue engineering (TE) aims to design new materials suitable for replacing/repairing damaged organs/tissues, thus avoiding a number of transplants or complex and expensive interventions [68]. The use of particular nanostructures can improve the biocompatibility of these materials, as well as recreate environments that mimic the native extracellular matrix, providing the mechanical, structural, and chemical–physical characteristics suitable to promote the biological interactions necessary to guarantee the compatibility of the scaffolds [69]. Among the metal oxides, ZnO is one of the most investigated for tissue-engineering applications, thanks to its antibacterial properties and to its role in promoting cell growth, proliferation, and differentiation [70]. These properties were experimentally studied for pure ZnO nanostructures [71] and in combination with composite materials, mainly polymers and ceramics [72,73], in order to realize 3D scaffolds manufactured using additive manufacturing techniques. ZnO NStr were tested to analyze their role in osteointegration processes. In this context, the selected nanomaterial must possess biomechanical properties to confer the restoration of the tissues, promote their growth, induce the formation of new bone, and guarantee the vascularization [71]. Park et al. [74] studied the in vitro, on MC3T3-E1 osteoblast, and in vivo osteointegration processes on two different ZnO-based nanostructures, i.e., a thin film and an array of nanoflowers, both grown on silicon substrates by pulsed laser deposition, and, in the second case, following photolithography. The promotion of osteointegration processes, as well as the antibacterial properties of ZnO nanostructures, makes them promising materials even in periodontal applications, such as dental materials and implants [75]. Memarzadeh and co-workers investigated a mixed coating of ZnO NPs and nanohydroxyapatite (NHA) on a glass substrate for the promotion of the growth of osteoblasts and antibacterial functions for possible applications in orthopedic and dental implants.

An essential requirement for TE is the biocompatibility of nanostructures in terms of cell viability and adhesion, as well as within the mechanisms involved in cell growth, proliferation, and differentiation processes. Ciofani and his collaborators [76] tested these properties on an array of ZnO NWs using two electrically excitable cell lines, namely, the PC12 cell line, which was suitable for modeling neuronal cells, and the H9C2 line, which was instead suitable for modeling muscle cells. With regard to differentiation, PC12 showed a well-developed neurite network, while H9C2 showed poor development of regular myotubes, presenting disordered dispositions, an aspect that was attributed to the different mechanical interaction between the cells and the substrate. Neuronal-type cells prefer a rather rigid substrate, such as the one used in this case; on the contrary, the muscle cells need a softer substrate for the correct fusion in myotubes.

Very recently Errico et al. [77] experienced a reversible myogenic–differentiation switching, effecting the functionalization of a glass substrate by means of a dense ZnO NWs array. The results of these studies suggested that, depending on the type of cell line, the ZnO NWs arrays can promote or inhibit cell differentiation.

The combination of inorganic components and organic matrices such as biopolymers improve the physicochemical properties, enabling them to satisfy the delicate balance between structure, biocompatibility, and stability [78]. Moreover, since the toxicity of ZnO nanostructures is concentration-dependent, the use of a methodology that incorporates the nanostructures within a matrix reduces their toxicity and increases the time required for their degradation.

Among the numerous synthesis techniques, electrospinning proved a promising approach for the production of hybrid polymeric nanoconstructs [79]. The basic set-up consists of a needle nozzle, a high-voltage power supply, a container for spinning fluid, and an electrode collector [80]. The electrospinning process depends on a number of parameters that can critically affect fiber formation and structure [81]. The study of interrelation between such parameters and nanofiber properties are considered very crucial for cell–scaffold interactions and cell growth. Depending on the cell type, specific electrospinning parameters have to be chosen for the achievement of optimal pore dimension, porosity, fiber diameter, and orientation [82].

Regarding the solution parameters, it is necessary that the concentration of the starting solution varies within a useful range. In fact, for concentrations below a minimum, a set of fibers and grains are obtained (beads), while, beyond a maximum concentration, it is impossible to maintain a constant flow at the level of the needle tip. The molecular weight influences instead the electrical and rheological properties and, therefore, the morphological characteristics of the fibers. In fact, the molecular weight reflects the number of bonds between the polymer chains in solution [29]. The selection of a desirable solvent is fundamental for the optimization of electrospinning. In fact, the surface tension depends substantially on the type of solvent and on the difference in solubility, while the viscosity of the solution and the high relative humidity can contribute to the formation of pores in the electrospun fibers [83,84]. Recent studies focused on the use of less toxic solvents for electrospinning, although the choice of these solvents requires an accurate optimization process [85].

Process parameters such as applied voltage, tip-to collector distance, type of collector, and the electric field have effects on the jet impact speed. The electrospinning process starts at a threshold voltage able to induce the polarization of the solution; the speed with which the syringe is fed influences the speed of the jet and the solvent evaporation process. Generally, low feed rates are more desirable since the solvent has more time to evaporate, whereas too high fluxes result in the formation of granular fibers due to the inadequacy of the achieved evaporation level [86,87]. A minimum distance between the tip and the collector is needed to allow the solvent to evaporate before it reaches the collector, thus avoiding the formation of unwanted granules in the final structure; moreover, the needle tip-to-collector distance has a considerable influence on the nanofiber diameter and the nanoweb collection zone [88].

Numerous biopolymers were used in combination with ZnO for tissue-engineering applications; among them, poly(ɛ-caprolactone) (PCL) has numerous advantages such as biocompatibility and biodegradability [89], and it is approved by the Food and Drug Administration (FDA) and used in clinical applications [90].

With regard to antibacterial and tissue regeneration properties, Bottino et al. [91] tested the potential application for periodontal regeneration of PCL/ZnO NPs and a PCL gel/ZnO NP electrospun scaffold. In particular, they studied the antibacterial properties of these composite materials against two known periodontal pathogenic bacteria: *Porphyromonas gingivalis* (Pg) and *Fusobacterium nucleatum* (Fn). They used 0.5, 15, and 30 wt.% ZnO, and they observed that, upon increasing ZnO content, antibacterial properties improved, but cell viability worsened, an aspect tested on human dental stem cells (hDPSCs). A good compromise was achieved using a 15 wt.% ZnO scaffold. An inhibition of bacterial activity was found, especially toward Fn; the PCL gel structure instead influenced the antimicrobial activity toward Pg. In particular, the presence of the gel changed the behavior of the scaffold from hydrophobic to hydrophilic, increasing the wettability of the fabric. The PCL gel also showed better mechanical properties in terms of tensile strength, Young’s modulus, and elongation at break.

An important property of the PCL/ZnO hybrid material lies in its electrical conductivity. Sezer and his group [92] explored this aspect for the regeneration of neuronal tissue. They used zero-valent zinc NPs at different concentrations (5, 10.15, and 20 wt.%) in solution together with PCL, making the material through electrospinning; they tested linear electrical conductivity, mechanical properties, the proliferation of U87 glioblastoma cells, and the toxicity on fibroblasts. The morphological properties of the fibers changed according to the Zn content, but a direct correlation between fiber diameter and Zn content was not identified. Regarding the mechanical properties, all the samples containing Zn had better values than the fibers containing only PCL. Electrical conductivity is a fundamental parameter for cells capable of being electrically stimulated, such as neuronal tissue cells; the results showed that the conductivity of fibers with 5 wt.% and 10 wt.% Zn was approximately equal to that of the nervous tissue. The authors emphasize the positivity of the results and conclude that further studies are needed to investigate the effect of the catalytic activity of Zn NPs on neuronal cells.

Augustine [93] and his group tested a PCL/ZnO composite scaffold focusing on the angiogenic mechanisms induced by commercial ZnO NPs loaded on an electrospun scaffold intended for TE. They used PCL with different percentages of ZnO NPs ranging from 0.5 to 4 wt.%. The scaffolds with 1 and 2 wt.% showed the best behavior both in cell proliferation tests in vitro, conducted on human dermal fibroblasts (HDFa), and in the test of chorioallantoic egg membrane (CAM), which showed the formation of blood vessels following the insertion of the scaffold. For this reason, the scaffold with 1 wt.% ZnO NPs was selected for the next subcutaneous implantation in guinea pigs for five days. During this test, the formation of mature blood vessels and a branched capillary network was demonstrated, as well as the migration of fibroblasts from the walls toward the inside of the scaffold. Furthermore, a circular arrangement of red blood cells was observed, indicating the beginning of an angiogenic process. Finally, the Western blot test showed that the main cause of angiogenesis activation was linked to the presence of small percentages of ZnO NPs that stimulated the production of proangiogenic factors, expressed by fibroblast growth factor-2 (FGF2) and vascular endothelial growth factor (VEGF) proteins.

Another interesting polymer for TE applications, thanks to its piezoelectric properties, is polyvinylidene fluoride (PVDF). Li and co-workers [94] analyzed PVDF and ZnO as potential bone TE materials. In their study, PVDF scaffolds doped with ZnO NPs (ZnO/PVDF) were prepared by electrospinning increasing ZnO concentrations and the ratio of the β-phase PVDF. The results showed an improvement of the elasticity modulus, elongation at break, and maximum load; in addition, piezoelectrically excited scaffolds exhibited much greater osteoblast density than control and compared to unexcited scaffolds, indicating that the piezoelectric ZnO/PVDF scaffolds can promote osteoblast proliferation through piezoelectricity. 

While the PVDF needs to be mechanically stretched to form the piezoelectric crystalline phase (beta phase), the co-polymer polyvinylidene fluoride–trifluoroethylene (PVDF–TrFE) instead possesses a permanent piezoelectric nature and does not need mechanical stretching before the poling. Its intrinsic electrical properties were studied for the enhancement of neuritis extension [95], to manipulate the fibroblast cellular behavior and proliferation. Augustine et al. [96] recently studied the biocompatibility of (PVDF–TrFE)/ZnO nanocomposite scaffolds in terms of cell adhesion and formation of blood vessels. The polymer was loaded with different percentages of ZnO, from 1 to 4 wt. %. In vitro cell cultures were made using human mesenchymal stem cells (hMSCs) and human umbilical cord endothelial cells. In vivo tests were performed on the Wistar rats, in which the formation of a highly branched capillary network of blood vessels was found. Moreover, in this study, the piezoelectric properties of the scaffold were taken into consideration, as a stimulating cause of a better cellular response. In fact, the electrical potential generated by the piezoelectric scaffold can convert the mechanical energy generated by the cellular environment into electrical signals that increase the cellular response. This aspect was highlighted by the Fourier-transform infrared spectroscopy (FTIR) analysis, which indicated a relative abundance of the electro-active β-phase of the nanocomposite material, compared to the net scaffold. 

In addition to the polymers already considered, numerous other biocompatible polymers were used, in combination with ZnO, in electrospinning processes aimed at producing materials for tissue regeneration. Amna et al. [97] produced a spider web using polyurethane (PU) and ZnO NPs. The particular bimodal structure, which alternated fibers with a larger diameter and very thin fibers similar to spider webs, was probably generated by the ionization of the polymeric solution in the presence of ZnO NPs. Furthermore, the presence of ZnO increased the overall crystallinity of the polymer. The same group [98] made one-dimensional ZnO-doped TiO_2_ by electrospinning using a colloidal gel composed of zinc nitrate, titanium isopropoxide, and polyvinyl acetate (PVA), which was subsequently annealed at 600 °C for 2 h. They used a standard Cell Counting Kit 8 (CCK-8) assay to study the effects of the material on adhesion, proliferation, and growth of C2C12 myoblasts. Balen et al. [36] produced a nanostructured composite of poly(methyl methacrylate) (PMMA) and ZnO NPs at concentrations of 0, 3, 5, 10, and 15 wt.%, using two different techniques: casting, to obtain a film, and electrospinning, to make a fibrous construct. They then studied the structural, thermal, and optical properties and the biocompatibility of the two materials. The results showed, in the case of fibrous material, that the ZnO content reduced the diameter of the fibers and the number of bids, as well as exhibiting greater hydrophobicity. For both categories, the ZnO improved the optical properties of the composite, with an intense absorption around 320 nm and a high luminescence in the ultraviolet (UV) region. Biological tests showed a better behavior of the material made with electrospinning, thanks to the greater surface area and its greater affinity and morphological similarity with the extracellular matrix; the fibroblast cells indeed showed greater vitality, further improved by the ZnO NP content. Percentages of ZnO higher than 1 wt.% increased the biocompatibility of the material; however, at 15 wt.% concentration, the cell proliferation was inhibited, due to the cytotoxic effect exerted by the ZnO NPs. Table 2 summarizes the main results obtained from the studies discussed in this paragraph, related to tissue-engineering applications.

### 6.2. Wound-Healing Applications

A hotspot application in the medical field is certainly represented by “wound healing” following trauma, surgical operations, implants, etc. A good wound dressing must be able to ensure an environment suitable for wound healing and must, therefore, guarantee a sufficient level of moisture, allow the exchange of gas, prevent the occurrence of infections caused by microorganisms, allow the removal of exudates, and minimize the scar formation. In addition, it must be non-toxic, non-allergenic, easily removable, and biocompatible [99]. Recently, the use of inorganic antimicrobials, including metal nanoparticles, gained considerable interest due to their broad antimicrobial spectrum, and their lower tendency to develop bacterial resistance [100,101]

The antibacterial and catalytic properties of ZnO and its biocompatibility make it an excellent candidate for wound-healing applications as it promotes regeneration and re-epithelialization of tissues and prevents scar formation [102].

**Figure 6:**
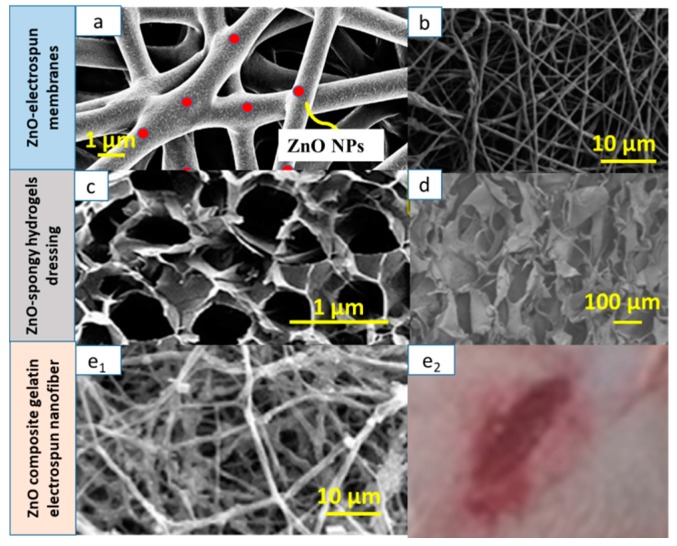
Wound-healing solutions. (**a**) Higher-magnification SEM micrograph of poly(ɛ-caprolactone) (PCL) membrane containing 1 wt.% ZnO nanoparticles, (**b**) SEM image of poly(3-hydroxybutyrate-co-3-hydroxy-valerate) (PHBV) reinforced with cellulose nanocrystal (CNC)–ZnO at weight loadings of 5%. (**c**) SEM image of keratin–chitosan-ZnO nanocomposite hydrogel. (**d**) SEM image of surface of chitosan (CS)–Ag/ZnO-1.0 (sponge immersed in 1.0 mg/mL of Ag/ZnO solution); (**e1**) SEM image of cefazolin-loaded zinc-oxide nanoparticle composite gelatin nanofiber, and (**e2**) dorsal skin region of rat embedded with the same nanofiber wound dressing.

Recent studies proposed various solutions that combine ZnO with different kinds of dressing in the form of gelatin/ointments [103], hydrogel [104,105,106], or electrospinning mats [107], made mainly with synthetic polymers or natural polymers or even a mix. Figure 6 shows a comparison between fibrous tissues manufactured by electrospinning, hydrogels, and electrospun gelatinous fibers.

Focusing on electrospinning, Shalumon and his research team [108] fabricated a sodium alginate (SA)/poly(vinyl alcohol) (PVA)/ZnO NP fibrous mat and studied the antibacterial properties toward *Staphylococcus aureus* (*S. aureus*) and *Escherichia coli* (*E. coli*) bacteria and the biocompatibility on L929 cells, proposing such a material for wound-healing applications. In addition, they analyzed the influence of ZnO on fiber properties such as viscosity, conductivity, and thermal stability. The results obtained showed that the insertion of the ZnO NPs had a slight effect on the viscosity and a more marked influence on the conductivity, which increased with the increase in ZnO content. The blend of SA/PVA was more stable to thermal decomposition when compared with the individual polymers, while thermal stability did not seem to be particularly enhanced by the presence of nanoparticles. Antibacterial studies demonstrated that the mats showed an inhibition zone in both bacteria for all ZnO concentrations, directly proportional to the ZnO concentration. Cytotoxicity studies indicated that fibers with 0.5 and 1 wt.% ZnO concentrations are less toxic, while cell viability decreased as the ZnO concentration increased. The authors concluded that there was a need to find an optimal concentration with the least toxicity while providing maximum antibacterial activity. 

Augustine and his collaborators [109] demonstrated the in vivo cell proliferations and wound-healing properties of an electrospun PCL/ZnO NP membrane. ZnO nanoparticle-embedded membranes did not show any significant sign of inflammation. A PCL membrane with 1 wt.% ZnO was implanted subcutaneously in a guinea pig’s dorsal cervical defect. The scaffold did not show any significant sign of inflammation and, compared to pristine one, had better proliferation, cellular vitality, adhesion, and growth of fibroblasts, which migrated from the subcutaneous regions toward the skin, promoting wound healing with wound closure without scarring. The C-reactive protein test (CRP), measuring the concentration of a protein produced by the liver in response to an inflammation/infection, by means of pig blood agglutination tests, also demonstrated the good properties of the selected scaffold.

Abdalkarim et al. [110] produced electrospun nanofibrous membranes from cellulose nanocrystal–ZnO (CNC–ZnO) nanohybrids as reinforcing materials in biodegradable poly(3-hydroxybutyrate-co-3-hydroxy-valerate) (PHBV). The incorporation of CNC–ZnO nanocrystals improved the uniformity and reduced the diameter of the PHBV nanofibers. With regard to mechanical properties, an improvement in tensile strength and Young’s modulus for nanofibrose membranes with 5.0 wt.% CNC–ZnO concentration was found. Furthermore, an increase in the initial decomposition temperature and the maximum decomposition temperature values was recorded. The nanofibrous membranes presented a positive effect on barrier properties and absorbency of simulated fresh blood. The 5.0 wt.% CNC–ZnO membrane showed the best antibacterial activity against *E. coli* and *S. aureus* bacteria. This nanocomposite hybrid material showed good results for potential use in antibacterial wound dressing.

Ahmed and his team [111] designed new chitosan/PVA/ZnO electrospun nanofibrous mats for diabetic wound healing. They demonstrated the antibacterial properties against *E. coli*, *Pseudomonas aeruginosa* (*P. aeruginosa*), *Bacillus subtilis* (*B. subtilis*), and *S. aureus*, which were better than those of fibers made with only chitosan/PVA; furthermore, the nanocomposite also had superior antioxidant properties. The authors also tested wound-healing skills in vivo on subcutaneous wounds in diabetes-induced rabbits (six months and weight of 0.8 to 1.3 kg). The results showed that chitosan/PVA/ZnO nanofibrous membranes resulted in accelerated wound healing. The mix between the biocompatibility and non-toxicity of PVA, the properties of chitosan (which promote a rapid contraction of wounds, and stimulate the proliferation of fibroblasts, the formation of collagen, and the deposition of hyaluronic acid in the vicinity of the wound), and the antibacterial and angiogenic properties of ZnO NPs made this material a good dressing for diabetic wounds. In conclusion, the authors stated that new studies to better understand the mechanism of action of this material, in addition to analyzing its genotoxicity, together with experiments on human subjects, will be necessary.

Rath et al. [112] combined the antimicrobial properties of ZnO NPs and cefazolin, a drug usually adopted for the treatment of post-operative wounds, exploiting both the fibrous morphology that can be obtained through electrospinning and the properties of gelatin, which is a bioavailable, economic polymer that presents good swelling and allows counteracting fluid losses due to exudation, improving wound healing. They manufactured cefazolin-loaded zinc-oxide nanoparticle composite gelatin nanofiber mats (both separately and in combination) for post-surgical operation wounds. Firstly, they determined the minimum inhibition concentration for cefazolin, ZnO NPs, and their mixture against *S. aureus*; then, they performed in vitro tests on the antibacterial properties and final in vivo experiments to evaluate the capacity of wound healing on Wistar rats. The results showed sustained drug release behavior and good antibacterial efficacy, especially for ZnO and cefazolin in a 1:1 weight ratio. From the in vivo tests, it was found that the hybrid material had more rapid and effective wound healing compared to the fibers loaded with drugs only or ZnO NPs only; moreover, the histological examinations revealed a greater cell adhesion, re-epithelialization, and production of collagen by the composite material. 

Kantipudi et al. [103] used AgNPs and Ag-ZnO composite NPs (0.1 g NPs) formulated into gel using the Carbapol 934 as a base gel, and they tested their ability to wound healing in vivo on excision wound (4 cm length and 2 mm depth) in adult male Albino Wistar rats. The composite showed good wound healing ability from the early stages, and after 10 days the wound showed fibrosis, caused by rapid epithelialization of the skin, indicating maximum effectiveness of healing. The wounds treated with only Ag NPs did not exhibit fibrosis, probably due to the insufficient antibacterial capacity compared to the composite material. Finally, in the case of using a standard dermazine drug, the healing process appeared very slow.

**Figure 7:**
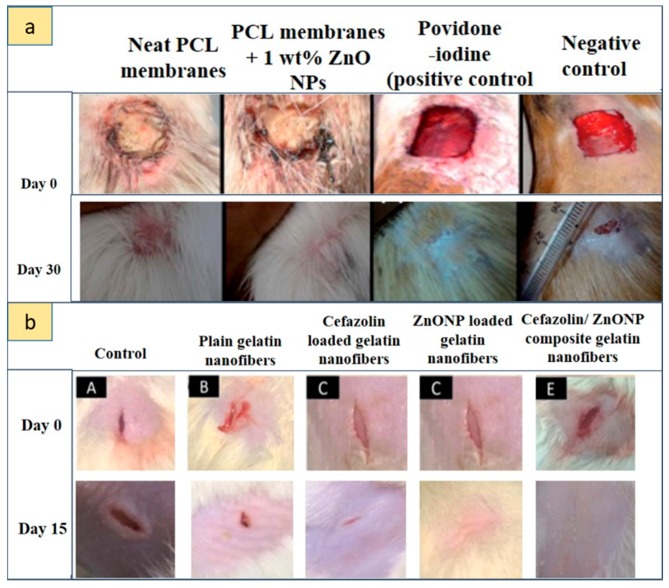
Results of different wound-healing dressing solutions in vivo. (**a**) Wound-healing activity of the membrane. The first column indicates neat PCL membranes, the second column indicates a PCL membrane incorporated with 1 wt.% ZnO NPs, the third column indicates povidone–iodine-treated wounds (positive controls), and the fourth column indicates negative controls. (**b**) Physical estimation of wound healing at various time intervals in the control (A), plain gelatin nanofibers (B), cefazolin-loaded gelatin nanofibers (C), ZnO NP-loaded gelatin nanofibers (D), and cefazolin/ZnO NP composite gelatin nanofibers (E) (adapted from [109], with permission from RSC, 2019, and from [112], with permission from Elsevier, 2019).

Figure 7 depicts the in vivo test results described in References [109,112], while Table 3 summarizes the main results with regard to wound healing. 

### 6.3. Antimicrobial Materials

**Figure 8:**
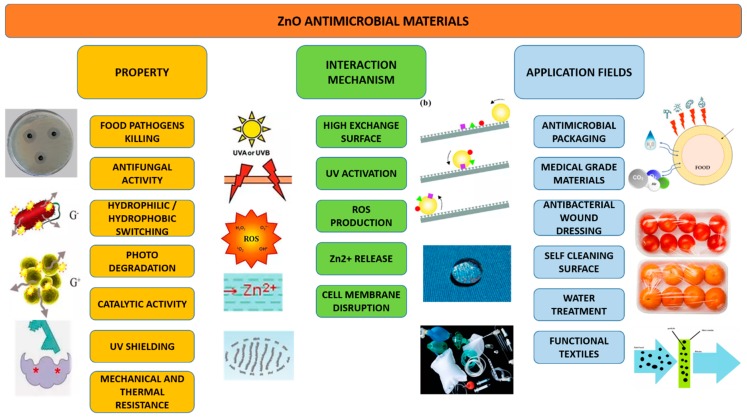
Properties, mechanisms of action, and applications of new ZnO-based antimicrobial materials.

The tendency to use metal compounds, including the ZnO-based ones, inside polymer matrices allowed creating new multifunctional materials with antibacterial and antifungal properties [113] to be used for a wide range of sectors, including photocatalytic materials for the degradation/removal of polluting species [114,115], materials for water treatment/separation [116], antiseptic and antibacterial membranes for the biomedical sector, food packaging, special self-cleaning fabrics [117], super-hydrophobic and antibacterial surfaces, and many others [118]. The confinement/anchoring of the nanostructures inside or on the surface of the polymers allows reducing the toxicity, optimizing the useful active concentrations, and creating a synergic effect between the properties of the different phases of the composite material obtained [119]. The antibacterial properties of ZnO can also be used for food storage and preservation; among the various solutions, one of the most promising involves the creation of an active antibacterial food packaging in which the material in contact with food is able to modify its characteristics and the environment that surrounds it [120,121]. The incorporation of ZnO inside polymeric matrices allows obtaining an active food packaging able to provide the proper antibacterial properties and to increase the mechanical and thermal properties of the packaging [122]. Figure 8 summarizes the properties of these new antibacterial ZnO–polymeric materials, the main antibacterial action mechanisms, and applications in which these materials are intended.

Electrospinning proved to be an effective and promising technique for the construction of these new antibacterial tissues with multifunctional properties, as described in the examples below. Wang et al. [123] created hierarchical nanofibers of electrospun polyamide-6 (PA-6) subsequently subjected to atomic layer deposition (ALD) and a hydrothermal method for the deposition and growth of ZnO “water lily”- and “caterpillar”-like NRs. They then studied the antibacterial properties against *S. aureus* and found that the caterpillar-like NRs had better antibacterial activities, as indicated by the larger diameter of the inhibition zone; however, further studies are needed to understand the contributions of ALD cycles and the hydrothermal reaction period to the antibacterial properties. 

The photocatalytic and optical properties of ZnO can be exploited to enhance the overall antibacterial effect. Prone and co-workers [124] recently developed mats with coaxial and uniaxial fibers of PCL and Zn-based NPs by electrospinning and studied their antibacterial properties toward *E. coli* and *S. aureus* under the action of UV-A light. They used different concentrations of ZnO NPs in the 9–25 wt.% range and selected this range because no significant inhibition of planktonic growth and biofilm bacteria was previously found at concentrations below 9%. During the electrospinning process, they studied the effect caused by the addition of ZnO NPs on the surface charge density of the jet and found that, as the concentration of ZnO increased, the stretching forces exerted under the action of the electric field increased, with a consequent reduction in diameter of the fibers. The Energy-dispersive X-ray spectroscopy (EDS) results demonstrated homogeneity in the distribution of NPs within the fibers, an important aspect to guarantee the uniformity of the antibacterial properties. Moreover, in the case of coaxial fibers, the NPs were mainly concentrated in the outer layer, due to the lower polarization of the jet’s inner core, an advantageous aspect to increase the antibacterial surface extension; in the uniaxial fibers, the NPs were instead mainly confined inside. The authors also demonstrated that the presence of ZnO NPs reduced the hydrophobicity of the tissue compared to the use of pristine PCL fibers; moreover, the ZnO NPs facilitated the degradation of PCL, reducing its crystallinity. The turbidity test showed that the nanocomposite exerted an inhibitory action of the planktonic growth caused mainly by the release of Zn^2+^ ions and by the photocatalytic oxidation process. In fact, if the tissue was illuminated with UV-A for 15 min before the inoculation of the bacteria, there was an increase in the photocatalytic production of ROS by the ZnO NPs, especially in the case of coaxial fibers. 

Anitha et al. [125] manufactured ZnO nanoparticle-embedded cellulose acetate (CA) fibrous membranes by electrospinning and tested their optical, antibacterial, and water-repellent properties. Also, in their work, the technique of electrospinning proved to be effective in avoiding the agglomeration of the particles and maximizing the active antibacterial surface. According to the results of other studies, the antibacterial action was stronger against *S. aureus* than toward *E. coli*, probably due to the different structural nature of the bacteria cell walls, which, in the case of *S. aureus*, is presented as a multilayer porous membrane of peptidoglycan and is, thus, more susceptible to intracellular transition of nanoparticles. However, antibacterial activity against *Klebsiella pneumoniae* (*K. pneumonia*) was not reported. With regard to the wettability tests, the results of the contact-angle measurements indicated that the properties of the CA passed from hydrophilic to hydrophobic due to the ZnO impregnation. The material was suitable for use as an antibacterial hydrophobic surface without the need for further surface treatments.

Kim et al. [126] developed, by electrospinning, polyurethane (PU) nanofibers coated with polydopamine (Pdopa) using a deep coating method and subsequently put them into a ZnO NP solution as a seed layer for the hydrothermal growth of ZnO NRs. The NR film obtained adhered firmly to the surface of the PU fibers, and the results showed excellent photocatalytic and antibacterial performances under the action of light-emitting diode (LED) devices with low UV intensity. The photocatalytic activity was investigated by monitoring the degradation of a blue methylene (MB) solution by measuring the absorbance via a UV–visible spectrophotometer. The authors speculated that this material, thanks in part to its high reusability and durability, may be suitable for developing antifungal photocatalytic membranes or for degrading organic pollutants and purifying wastewater.

Malwal and Gopinath [127] synthesized CuO–ZnO composite nanofibers using electrospinning and subsequent calcination for water treatment applications; they then measured their antibacterial properties, absorption kinetics, absorption isotherm, and diffusion characteristics. The combination of antibacterial and absorption properties made this material suitable for water treatment and purification processes.

Liu and co-workers [128] fabricated electrospun nanofibers starting from ethylcellulose/gelatin solutions containing various concentrations of ZnO NPs. The presence of ZnO as a filler allowed increasing the hydrophobicity of the tissue, the water stability, and the antibacterial action toward *E. coli* and *S. aureus*, especially after UV irradiation. The authors stated that, thanks to these properties, the material could potentially be used in food packaging. Table 4 offers a summary of these results.

## 7. Conclusions

The toxicity of Zno NStr strongly depends on their physical, chemical, and morphological (shape, size) properties. Toxicity is typically concentration- and time-dependent, and there is a threshold value below which the use of ZnO nanostructures does not appear to compromise cell viability. This value is lower for smaller, spherical, and higher-aspect-ratio ZnO NPs, while it increases for nanostructures such as ZnO NRs. In fact, as the nanostructures become smaller and more reactive, with a high surface-to-volume ratio, the cell uptake increases. Solubility at different pH and aggregation phenomena are other parameters that influence cytotoxicity. Among the main causes, the production of ROS, zinc ion release, breakdown of the cell membrane, impairment of mitochondrial functions, DNA damage, and the activation of apoptosis and necrosis processes were highlighted. The selectivity of ZnO NStr toward malignant and non-malignant cell lines makes them interesting for cancer therapy applications. The in vivo studies, although they are still limited, and although the administration of nanostructures is at higher concentrations than the clinical ones, confirmed the results obtained in vitro and showed that the most compromised organs are the kidneys, liver, spleen, pancreas, lungs, reproductive system, and the brain. In conclusion, it is necessary to carry out a careful study of the doses and times of administration of the nanostructures, as well as choosing the most suitable shape and size according to the target application, for an effective and safe use of these nanomaterials. Electrospinning is able to create macroscopic textile materials and coatings that inherit the properties of the constituent ZnO NStr. Clearly, the optimum in terms of concentration and type has to be determined vs. performance in terms of biological targets by means of rational design tools such as applied statistics and experiment design. Applications in wound healing and antibacterial barriers enabled by electrospinning promise to be particularly disruptive. 

## Figures and Tables

**Table 1:** Description of the systems analyzed in the graphic reconstruction of Figure 5. MTT, 3-(4,5-dimethylthiazol-2-yl)-2,5-diphenyltetrazolium bromide assay; L/D, live/dead viability/cytotoxicity assay; WST, Cell Counting Kit 8 assay; NR, neutral red assay; TB, trypan blue assay.

Ref	C0	Well Type	Cell Line	ZnO NStr Type	ZnO NStr Dimensions	Cell Viability Assay
[41]	1 × 10^5^ cells/well	96-well plates	T-cell	NPs	4–20 nm	L/D
[41]	5 × 10^4^ cells/well	96-well plates	Jurkat	NPs	4–20 nm	L/D
[42]	5 × 10^4^ cells/well	12-well culture dish	BEAS-2B	NPs	~10 nm	WST
[53]	-	96-well plates	HMMs	NWs	120 nm × 2–5 µm	NR
[44]	5 × 10^4^ cells/cm^2^	Flat 96-well plates	NHDF	TRPs	~37 nm	MTT
[62]	1 × 10^4^ cells/mL in each well	96-well plates	Mouse podocytes	NPs	20–80 nm	MTT
[61]	1 × 10^4^ cells/well	96-well plates	LCs	NPs	70 nm	MTT
[61]	1 × 10^4^ cells/well	96-well plates	SCs	NPs		MTT
[47]	1 × 10^4^ cells/well	96-well plates	HEK 293	NPs	25–40 nm	NR
[64]	1 × 10^4^ cells/well	96-well plates	A549	NCs	4.7 ± 0.8 nm	MTT
[64]	1 × 10^4^ cells/well	96-well plates	MRC-5			MTT
[46]	1 × 10^5^ cells/well	96-well round-bottom plates	FaDu	NPs	20 nm	MTT
[46]	1 × 10^5^ cells/well	96-well round-bottom plates	BMSC	NPs		TB
[65]	10^4^ cells/mL	96-well plates	HeLa	NPs	~50 nm	WST
[55]	1 × 10^4^ cells well	96-well plates	A549 (2)	NRs	diameter ≈ 52 nm	MTT

**Table 2:** Main results of recent studies on ZnO-based nanomaterials and electrospun ZnO–polymeric hybrid nanomaterials for tissue-engineering applications.

Type of System	Ref	Description of the System	ZnO Concentration	Cell Line/Bacteria	In Vivo Experiments	Main Results
ZnO NStr/ZnO array for experimental purposes	[74]	ZnO NFls arrays on Si substrate	Zinc nitrate solution 25 mM	MC3T3-E1 osteoblast culture	Implantation on calvarial bone defects of Sprague Dawley rats	Formation of lamellipodia and filopodia
	[74]	ZnO NWs arrays incubated with a collagen solution		PC12 and H9C2	_	Adhesion, proliferation, and differentiation of two different electrically excitable mammalian cell lines
	[77]	ZnO NWs arrays on a glass substrate		Mesoangioblasts	_	- Reversibly locked differentiation - No cell damage - Differentiation capabilities completely recovered upon cell removal from the nanowire substrate and re-plating on standard culture glass
ZnO/PCL electrospun scaffold	[93]	PCL+ZnO NPs	0.5–6 wt.%	HDFa	Implantation in guinea pigs	- Proangiogenic properties of ZnO/PCL fibers - Increase in the formation of mature blood vessels and highly branched capillary network
	[91]	PCL and PCL/gelatin + ZnO NPs	0, 5, 15, 30 wt.%	Pg, Fn, hDPSCs, AllCells LLC, Alameda, CA.	_	- Potential application in periodontal regeneration - Good antibacterial properties
	[92]	PCL matrix + zero-valent Zn NPs	5, 10, 15, 20 wt%	Neuroglioblastoma cells, human primary fibroblasts	_	Small concentrations of Zn NPs promoted neuronal cell proliferation with relative non-toxicity for fibroblasts
ZnO–polymeric (other polymers) electrospun implantable scaffold	[97]	ZnO–PU scaffold	5 wt.%	mouse fibroblast	_	Fibroblast viability, adhesion, and proliferation
	[96]	(PVDF–TrFE) + ZnO NP scaffold	0, 0.5, 1, 2, 4 wt.%	Red blood cells, White blood cells, platelet, hMSCs), HUVECs	Subcutaneous implantation in Wistar rats	- Tissue regeneration due to the piezoelectric properties of the composite components - Biocompatibility of the system in vitro - Angiogenic properties in vivo
	[94]	β-phase PVDF + ZnO NPs	0.5, 1, 2 mg/mL	Human osteoblasts, *S. aureus*, methicillin-resistant *S. aureus*, *E. coli*.	_	- Improvement of the elongation modulus at break and load stress - Greater osteoblast density and antibacterial properties of the piezoelectrically excited scaffold
	[98]	1D ZnO-dopedTiO_2_ fabricated using colloidal gel	1 and 10 μg/mL of ZnO/TO_2_	C2C12 myoblast cells	_	Beneficial effect on the adhesion, proliferation, and growth of myoblasts
	[36]	PMMA + ZnO NPs fibers and films	0, 1, 3, 5, 10, 15 wt.%.	Fibroblast cells (L929)	_	- Good proliferation of fibroblast cells - Thermal stability - Luminescence with emission in the near-UV range

**Table 3:** Main results of recent studies on ZnO-based nanomaterials and electrospun ZnO–polymeric hybrid nanomaterials for wound-healing applications.

Type of System	Ref	Description of the System	ZnO Concentration	Cell Line/Bacteria	In Vivo Experiments	Main Results
Electrospun fibrous membranes	[108]	Sodium alginate/poly(vinyl alcohol) fibrous mat + ZnO NPs	0.5, 1, 2.5 wt.%	L929 fibroblasts cells, *S. aureus*, *E. coli*		- Fibers with 0.5 and 1% ZnO concentrations are less toxic - Inhibition for both the bacteria - Toxicity increase at the high ZnO concentration.
	[109]	PCL + ZnO NPs	1, 2, 4 wt.%		Membranes implanted subcutaneously in guinea pigs	- ZnO enhanced the cell adhesion, migration, and proliferation -No significant sign of inflammation - In vivo implant enhanced the wound healing without any scar formation
	[110]	Cellulose nanocrystal (CNC)–ZnO in poly(3-hydroxybutyrate-co-3-hydroxy-valerate)	CNC–ZnO suspension at 0, 3, 5, 10, 15 wt.%	*E. coli* and *S. aureus*		- Improvement in tensile strength and in Young’s modulus - High thermal stability -Good antibacterial activity
	[111]	Chitosan/PVA/ZnO NP nanofibrous membranes		*E. coli*, *P. aeruginosa*, *B. subtilis*, *S. aureus*	Subcutaneous wounds in diabetes-induced rabbits	- High antibacterial and antioxidant potential - ZnO accelerated wound healing in vivo
Spongy hydrogels	[104]	Ag/ZnO into chitosan sponge	Immersion in 0.1, 0.2, 0.5, and 1.0 mg/mL of Ag/ZnO and in 0.5 mg/mL of ZnO solution	*S. aureus*, *E. coli*, *P. aeruginosa*, human normal hepatocyte (L02)	BALB/c mice: wound with a length of 7 mm on the back	- Evaluation of the porosity, swelling, blood clotting, and in vitro antibacterial activity -Low toxicity in vitro - Enhanced wound healing, re-epithelialization, and collagen deposition in vivo
	[105]	Hydrogels of heparinized PVA/chitosan/ZnO NPs		Mouse fibroblast cells (L-929), *E. coli*, *S. aureus*		- Heparin release rate decreased by adding ZnO NPs - Good antibacterial protection of wounds
	[106]	Porous keratin–chitosan/n-ZnO hydrogel	ZnO nanopowder 0, 0.5, 1 wt.%	fibroblasts cells (NIH 3T3), *E. coli*, *S. aureus*	Sprague-Dawley rats: skin wound of 1.5 cm^2^ in the dorsum of the rat	- Biocompatibility in vitro. - Increased wound curing in vivo with quicker skin cell construction and collagen development
Gel and gelatin nanofibers or ointments	[112]	Cefazolin + ZnO NPs electrospun gelatin nanofiber mats	1:1 *w/w* combination of cefazolin and ZnO NPs (1–64 μg/mL)	In vitro release studies + antibacterial property for *S. aureus*	Wistar rats: 2-cm-long incision	- Therapeutic approaches for post-operative wound - Determination of minimum inhibitory concentration - Hybrid antibacterial nature of ZnO NPs and cefazolin - Accelerated wound healing
	[29]	AgNPs and Ag–ZnO NPs formulated into gel using Carbapol 934	0.1 g of NPs		Adult male albino Wistar rats: excision wound (4 cm length and 2 mm depth)	- Wound-healing properties of Ag–ZnO NPs in vivo - Rapid healing within 10 days when compared with pure AgNPs and standard drug dermazin

**Table 4:** Main results of recent studies on ZnO-based nanomaterials and ZnO–polymeric composite nanomaterials for sustainability applications.

Type of System	Ref	Application	Description of the System	ZnO Concentration	Cell Line/Bacteria	Main Results
ZnO Coating	[120]	Antimicrobial activity against food pathogens	ZnO (ZnO nanoparticle suspension)-coated Polyvinyl chloride film	93.75 and 187.5 ug/cm^2^	*E. coli*, *S. aureus*, fungal *Aspergillus flavus* and *Penicillium citrinum*	- Antimicrobial activities of Polyvinyl chloride-based films to inactivate food pathogens - Effective antibacterial activity for *S. aureus* - No antifungal activity
	[127]	Advanced functional textile	ZnO NP-coated polyvinylsilsesquioxane (PVSQ) composite	0, 0.3, 0.5, 1, 2, 3 g	*E. coli* and *S. aureus*	- Excellent UV shielding and stable superhydrophobic properties - Enhanced mechanical properties and thermal stability - Larger resistivity of the *E. coli* compared to the *S. aureus*
Electrospun nanocomposite membranes	[125]	Hydrophobic–bactericidal materials	ZnO NPs embedded on CA fibrous membrane	0.2 mol of zinc acetate dihydrate	*Staphylococcus aureus*, *E. coli*, *Klebsiella pneumoniae*, *Citrobacter freundii*	- Hydrophobic nature of the surface - Strong antibacterial activity
	[123]	Antibacterial application	PA-6 nanofiber modified with ZnO using ALD + hydrothermal reaction	100–150 cycles of ALD with ZnO seed layers (14.6 nm)	*S. aureus*	Efficient in suppression of bacteria survivorship
	[127]	Removal of biological/organic contaminants for water treatment and purification	CuO–ZnO–PVA nanofibers	50, 100, 150, 200, 250, 300, 350 ug/mL	*E. coli* and *S. aureus*	- Enhanced adsorption efficiency and antibacterial properties - Excellent adsorption capacity for congo red dye
	[126]	Photocatalysis and antimicrobial activity for organic pollutant degradation and waste water purification	Hierarchical ZnO NR deposited on PU nanofiber		*E. coli*	- High photocatalytic/antimicrobial activity at the low-intensity UV LED device with good reusability - Measure of the degradation of the methylene blue (MB) solution
	[124]	Antibacterial nanocomposite wound dressings	ZnO NP–PCL uniaxial or coaxial fiber structure	ZnO NPs 9, 12, 15 and 25 wt.% relative to PCL	*E. coli*, *S. aureus*	- Inhibition of planktonic and biofilm bacterial growth - Increased antibacterial properties for coaxial fibers and for exposure to UV-A light prior to bacteria inoculation
Gelatin composite films	[121]	Active shrimp packaging	ZnO NRs/clove essential oil incorporated into type B gelatin composite films	NR loading concentration 2% *w/w* of gelatin	*Listeria monocytogenes* and *Salmonella typhimurium*	- Film with low flexibility and high mechanical resistance- Oxygen and UV barrier property increased with ZnO NR incorporation - Composite films loaded with 50% clove essential oil and with ZnO NRs showed maximum antibacterial activity
